# No effects of GSM-modulated 900 MHz electromagnetic fields on survival rate and spontaneous development of lymphoma in female AKR/J mice

**DOI:** 10.1186/1471-2407-4-77

**Published:** 2004-11-11

**Authors:** Angela M Sommer, Joachim Streckert, Andreas K Bitz, Volkert W Hansen, Alexander Lerchl

**Affiliations:** 1 School of Engineering and Science, International University Bremen, Research II, Campus Ring 6, D-28759 Bremen, Germany; 2 Chair of Electromagnetic Theory, University of Wuppertal, D-42097 Wuppertal, Germany

## Abstract

**Background:**

Several reports indicated that non-thermal electromagnetic radiation such as from mobile phones and base stations may promote cancer. Therefore, it was investigated experimentally, whether 900 MHz electromagnetic field exposure influences lymphoma development in a mouse strain that is genetically predisposed to this disease. The AKR/J mice genome carries the AK-virus, which leads within one year to spontaneous development of thymic lymphoblastic lymphoma.

**Methods:**

320 unrestrained female mice were sham-exposed or exposed (each n = 160 animals) to GSM like 900 MHz electromagnetic fields for 24 hours per day, 7 days per week, at an average whole body specific absorption rate (SAR) value of 0.4 W/kg. Animals were visually checked daily and were weighed and palpated weekly. Starting with an age of 6 months, blood samples were taken monthly from the tail. Animals with signs of disease or with an age of about 46 weeks were sacrificed and a gross necropsy was performed.

**Results:**

Electromagnetic field exposure had a significant effect on body weight gain, with higher values in exposed than in sham-exposed animals. However, survival rate and lymphoma incidence did not differ between exposed and sham-exposed mice.

**Conclusion:**

These data do not support the hypothesis that exposure to 900 MHz electromagnetic fields is a significant risk factor for developing lymphoma in a genetically predisposed species, even at a relatively high exposure level.

## Background

The use of mobile phones is increasing worldwide, although electromagnetic fields emitted by mobile phones and base stations are a source of great concern. However, so far it is unclear, if non-thermal exposure has a direct influence on public health. French et al. [1] developed a theoretical model, by which radiofrequency radiation from mobile phones could induce cancer, via the chronic activation of the heat shock response. Non-thermal exposure to electromagnetic fields can result in an increased expression of heat shock proteins (hsp) [2,3]. This is a normal defense response to cellular stress. However, chronic expression of hsp is known to induce or promote oncogenesis, metastasis and/or resistance to anticancer drugs [1]. Additionally, 72 hours exposure of human lymphocytes to continuous 830 MHz electromagnetic fields caused a linear increase in chromosome 17 aneuploidy with rising specific absorption rates (SAR: 1.6–8.8 W/kg). This is a signal for genetic instability and may thereby lead to cancer development [4]. In principal agreement, few epidemiological studies suggest a relationship between the use of mobile phones and uveal melanoma [5] or malignant brain tumors [6-9] However, the overall literature does not provide persuasive epidemiological evidence that mobile-phone-related emissions are carcinogenic, although mobile phones have not been in use long enough to exclude long-term impact on health [10].

It was suggested that non-thermal exposition to high-frequency electromagnetic fields may rather have a tumor promoter than an initiator effect [9], since DNA, generally, does not appear to be significantly altered or damaged by electromagnetic fields [11]. In this respect it was discussed, if a possibly reduced excretion of the oncostatic hormone melatonin by electromagnetic fields may facilitate the development of estrogen dependent tumors [12]. However, the results of different rodent studies concerning tumor promotion are not consistent. On the one hand, no difference in radiation or chemically induced tumor growth could be found after long-term exposure to electromagnetic fields (860–900 MHz) in rats or mice [13-15] Additionally, exposure of human leukaemia cells to electromagnetic fields failed to induce any changes in apoptosis, micronucleation or differential gene expression [16]. On the other hand, long-term exposure to pulse-modulated electromagnetic fields similar to those used in digital mobile telecommunication significantly increased in one study the incidence of lymphoma in Eμ-*Pim*1 transgenic mice [17], which are genetically predisposed to develop lymphoma spontaneously, but not in another [18].

The differences between the studies may indicate that various species or strains as well as cancer types differ in their sensitivity to electromagnetic field exposure. The sensitivity to electromagnetic fields may result from an acquired lower resistance against adverse effects or a genetic predisposition [19]. Different proportions of a sensitive subpopulation within an epidemiological or experimental study would influence the interpretation of a possible role in carcinogenesis. However, national or international thresholds for electromagnetic field intensities must ensure adequate health protection also for susceptible people.

AKR mice are widely used in cancer research for their high leukaemia incidence (60–100%) [20]. Mice of this strain are viremic from birth and express in all tissues the retrovirus AKV, which is associated with spontaneous leukaemia development in mice [21-23]. Generally, leukaemia induced by a given virus is restricted to a single histopathological type; most common is a lymphocytic leukaemia originating in the thymus. However, the type of leukaemia induced can sometimes be altered by age or experimental manipulation [24,25]. Using AKR mice, we studied the incidence of tumors and survival rates under chronic influence of high-frequency electromagnetic fields. Despite some physiological differences between mice and humans, a good correlation between known or assumed human carcinogens and test results in rodent studies has been described, often with the same organs affected in humans and in rodents [26]. Therefore, the results of this study shall help to evaluate a possible health risk of mobile phones.

## Methods

### Animals and animal husbandry

320 female AKR/J mice were airfreighted from the Jackson Laboratory (Bar Harbor, ME, USA), at an age of 4–5 weeks. After arrival, animals were randomized and housed in groups of 6 or 7 on softwood bedding (Altromin, Lage, Germany) in polycarbonate cages (40 × 25 × 15 cm, W × L × H, Ebeco, Castrop-Rauxel, Germany), enriched with paper. Mice were allowed free access to mice standard food pellets (type 1324, Altromin) and tap-water. Twice a week cages were cleaned and water changed. Temperature was controlled at 21°C ± 2°C. The light was on a 12 hours light-12 hours dark cycle, with light on at 8 am. No sterilization measurements were taken, since AKR/J mice are not especially sensitive for pathogens. However, to prevent a possible transfection from humans to mice or mice to mice, respectively, masks and gloves were worn, which were sterilized between handling of different cages. Animals were inspected daily for signs of moribundity and were weighed (accuracy ± 0.1 g) and palpated weekly. Starting with an age of 6 months, blood samples were taken monthly from the tail. A tattoo in the ear allowed individual identification. The Bremen state commission for animal welfare according to §8a of the German animal welfare legislation approved the experiments (522-27-11/2-0).

### Exposure setup

In order to accomplish whole body exposure of a large number of non-restrained animals, one approach is to design exposure setups based on radial waveguides. Inside the waveguide the animals are kept in cages which are arranged at a constant distance from a radiating antenna in the centre, thus uniform exposure of the cages can be achieved. Another advantage is that the radial waveguide is an electromagnetic shielded system, on that score no costly shielding of any laboratory is necessary. The height of the waveguide is preferably chosen smaller than half a wavelength [28]. Thereby, single-mode operation of the TEM-mode is possible and by this homogeneous field distribution inside every cage can be guaranteed. However, in the actual study the plate distance of the radial waveguide must be chosen larger than half the wavelength of the exposure frequency due to the prescribed height of the cages of about 16 cm. Consequently, higher order modes are able to propagate in addition to the TEM-wave, which have inhomogeneous field distributions in the waveguide's cross-section. Furthermore, the simultaneous propagation of several modes leads to interference effects and thus to an unstable exposure field.

**Figure 1:**
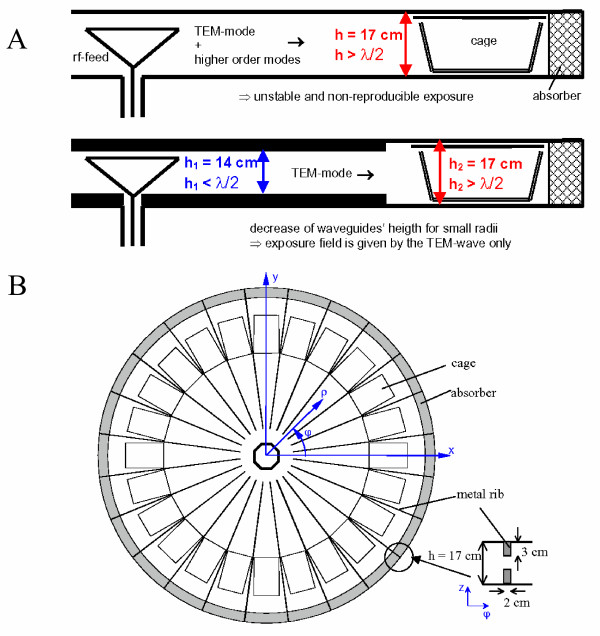
**Measures to guarantee stable and reproducible exposure fields. **a) Single mode excitation of the cage region b) Metal ribs used to shift the cut off frequency of unwanted higher order modes (e.g. TEz01, TMz01) out of the operation frequency range

Therefore, special modifications of the fundamental geometry of radial waveguides had to be performed to avoid the propagation of the unwanted higher order modes. In order to produce a well defined field distribution the cage region was excited only by the fundamental TEM-mode in a first step, i.e. for small radii the plate distance was kept below 14 cm and for larger radii the height was increased to the required value of 17 cm (Figure 1a). Since it was still possible that the additional modes were excited by the field scattered of the mice, metal rib structures were attached to the upper and lower plate between the cages in order to shift the cut off frequencies of these unwanted modes to higher values, so that they could not propagate at the exposure frequency (Figure 1b). The optimum size of the ribs was determined by numerically solving the eigenvalue problem of the modified waveguide. It yielded that a maximum attenuation of the higher order modes is reached for specific heights and widths of the ribs. Therewith, it was shown that the unwanted modes can be forced to become evanescent and thus a stable field distribution is achievable. Moreover, the rib structure does not alter the propagation constant of the TEM-mode, and the perturbation of the field distribution of the fundamental mode is negligible within the cage volume.

**Figure 2:**
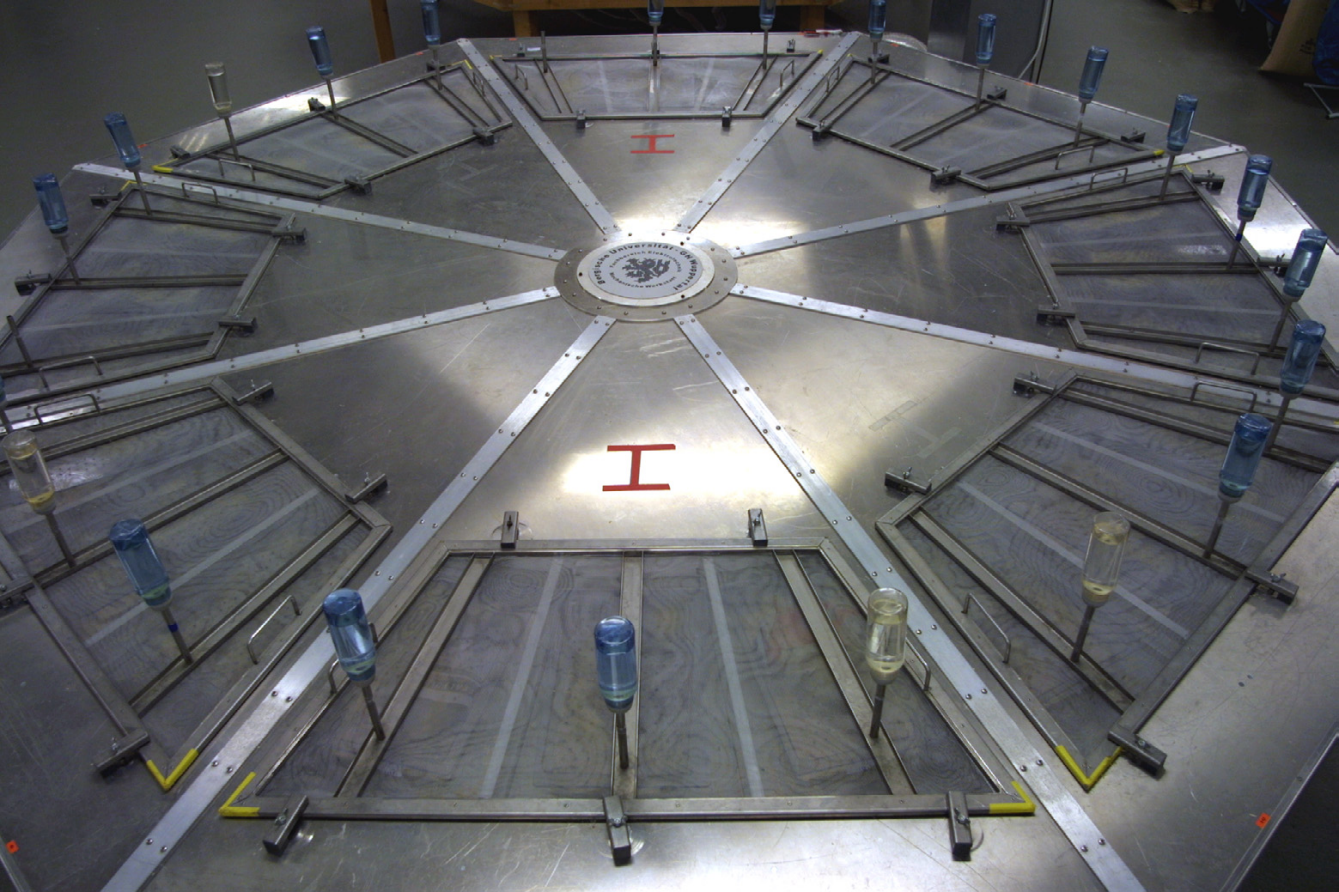
**One of the two identical exposure units viewed from top. **From an antenna in the middle (under the central plate; not visible in this picture) electromagnetic fields were fed into the radial waveguide and absorbed by either the animals in the cages or by absorbers at the boundaries of the waveguides. Each of the upper segments had a trapezoidal opening to allow for removing and inserting the cages. The openings were closed with wire mesh lids in order to guarantee penetration of light and gases and to prevented high-frequency electromagnetic radiation to leave or enter the exposure unit. Water bottles were placed outside the waveguides to prevent unwanted absorption.

**Figure 3:**
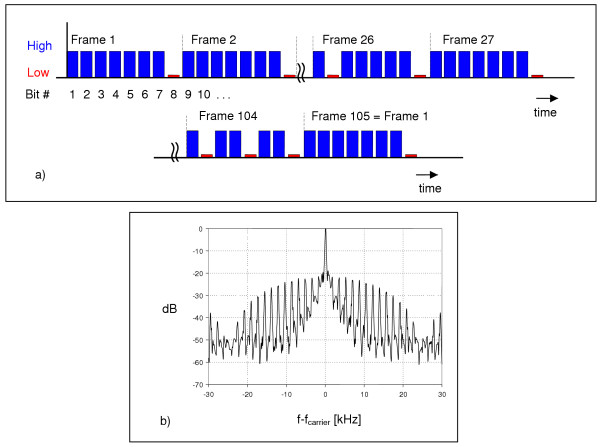
**Synthetic GSM modulation pulse pattern and frequency spectrum. **The exposure frequency was 900 MHz with a modulation simulating a typical GSM (Global System for Mobile Communication) scenario, namely a situation combining the emissions from a base station (downlink) with those from an uplink operating mobile phone, thus including 2.1 Hz, 8.3 Hz, 217 Hz, and 1733 Hz frequency components.

Both implemented waveguides (one for exposure, one for sham-exposure) of ca. 4 m diameter and 17 cm vertical plate distance were placed within the same room and carried up to 24 cages measuring 425 mm × 265 mm × 160 mm (L × W × H) each of them housing 6–7 mice. The cage area was covered with trapezoidal lids (3 cages per opening) with wire mesh (Figure 2). This design ensured easy maintenance and also that both light and gas could penetrate the lids while electromagnetic radiation was effectively shielded. At the outer boundaries of the units, absorbers were installed which caused minimal reflection and "hot spots". A signal generator (SMT 06, Rohde & Schwarz, Munich, Germany) and an amplifier (HLV-500, BEKO, Munich, Germany) were connected to the cone antenna of one unit via a "black box" so that it could not be seen which group of animals was exposed (blind design). The signal of the generator was modulated (BS 825F, BUGH Wuppertal, Germany) in a way which simulated a situation near a mobile communication base station (downlink) combined with a contribution from an mobile phone DTX operation mode (uplink), thus including 2 Hz, 8 Hz, 217 Hz, and 1733 Hz frequency components (Figure 3). For additional details see also [27]. Animals were exposed 24 hours per day with the exception of approximately 1 hour weekly when animals were weighed and palpated, and during which the cages were cleaned.

**Figure 4:**
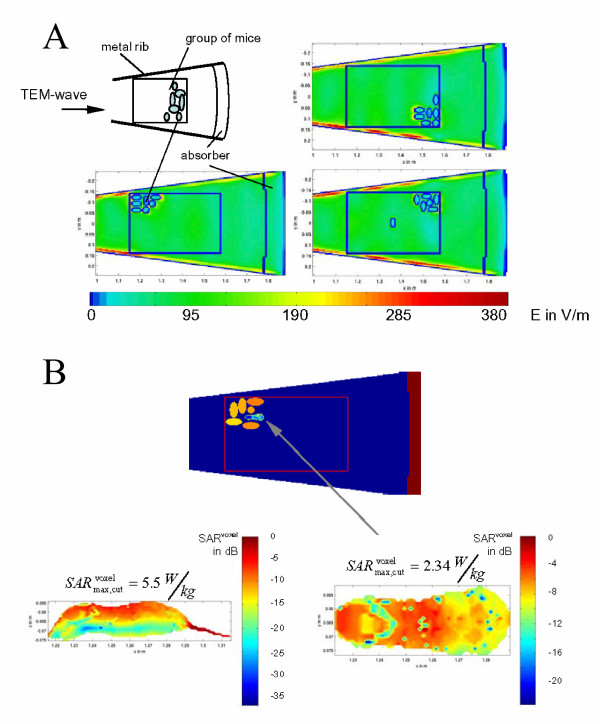
**Dosimetry. **a) Examples for groups of 7 mice inside a cage (P_in _= 35 W): Electric field distribution for a 15° sector of the radial waveguide. Higher field values occur due to the field concentration at the metal ribs between the cages (cf. fig. 1b). b) Example for insertion of an anatomical mice model into the group of ellipsoids: Localized SAR distribution (SAR_wb _= 366 mW/kg, resolution: 1.2 mm (voxel size)). The overall maximum of SAR_voxel _= 5.9 W/kg is not located in the cuts shown here.

The experiment was performed at a mean value of 0.4 W/kg of the whole body specific absorption rates. This value, which was stipulated by the financial backer, is five times higher than the limit of whole-body exposure for the general population and is based on the limit value for occupational exposure [29]. Since the mice can move freely, the whole body SAR varies with their postures and positions inside their cage. Therefore, the specific absorption rates in the mice were analyzed by numerical computations of the electromagnetic field distribution inside the radial waveguide for five different configurations of the animals, which were assumed to be uniformly distributed in time. Configurations account for groups of mice in the front and rear section of the cage as well as mice with head, left/right side toward the incident wave and upright posture. Since only the variation of the whole body SAR is subject of interest, it is sufficient to use simple homogeneous models (ellipsoids, length 6 cm, diameter 3 cm, appr. 32 g) filled with muscle tissue for the mice (Figure 4a).

After evaluation of all absorption rates per rodent it turned out that the standard deviation of the whole body SAR was ± 40%. The assessment of maximum localized SAR was performed by use of an anatomical mice model which was placed into the group of ellipsoids. (Figure 4b).

The required time-averaged input power of the exposure unit was 35 W. The presence of the field was monitored continuously, again via a "black box". In Streckert et. al [30] features of the exposure facility, which was previously used for experiments with rats, are described in detail.

Noise levels provoked by the integrated ventilation system were measured in close proximity to both units and were found to be identical (sound meter model 2218 with 1/3 octave filter set model 1616, Brüel & Kjaer, Naerum, Denmark). The total level was 69 dB (lin), and less than 25 dB at frequencies between 8 and 40 kHz. Thus possible disturbing effects of ultrasound were excluded.

### Pathology

Animals were sacrificed by CO_2 _gas when signs of a developing disease became evident or at an age of about 46 weeks, after a last blood sample was taken. A gross necropsy was performed focusing on main tissues of disease involvement (spleen, thymus and lymph nodes) and tumor infiltration (liver, kidney, lung, brain). Tissues were immersion-fixed in Bouin's solution for up to 24 h and subsequently in ethanol (70%), embedded in paraffin and sectioned at 5 μm. Blood smears were stained with Pappenheim's stain and tissue slices using hematoxylin and eosin. When a mouse was found dead in its cage (5 exposed, 7 sham-exposed mice) a necropsy was performed, but no tissues were fixed.

### Statistics

Group mean body weights were tested for a possible exposure influence in dependence of time, using multiple regression analysis (InStat 3.05, GraphPad). An unpaired t-test was applied to compare the loss of weight associated with lymphoma development. Survival curves and lymphoma incidence were plotted according to the method of Kaplan and Meier. Differences between curves were compared using the logrank test, with animals censored, which were still alive at the end of the study (Prism 4.01, GraphPad). Two-way ANOVA was used to test for possible changes in differential leucocytes counts with time and exposure.

Statistical significance of differences was tested two-sided at the p ≤ 0.05 level. If not indicated otherwise, data are given as means ± standard error of the mean. The exposure code was broken only after completion of the analyses.

## Results

### Body weight and water consumption

**Figure 5:**
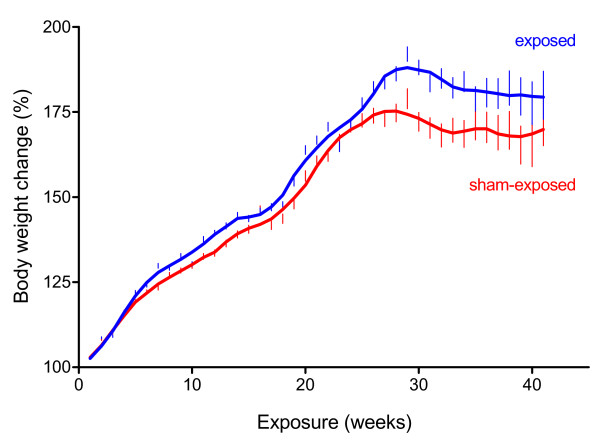
**Growths curves for female AKR/J mice exposed or sham-exposed to 900 MHz electromagnetic fields. **The relative weight increase of female AKR/J mice was more pronounced in exposed than in sham-exposed animals. Therefore, the mice's tendency to obesity seems not only to be strain-specific but was also significantly (p < 0.001) related to the exposure. Data are given as % of the animals weight (with 100%) at the beginning of the study ± standard error of the mean, n = 160 at the beginning of the experiment.

Chronic exposure to 900 MHz electromagnetic fields had no influence on the absolute body weight of female AKR/J mice; however, its influence on the relative weight change was significant (p < 0.001) (Figure 5). The rapid development of lymphoma in this strain of mice was associated with a loss of individual body weight of about 9.2% in exposed and 8.5% in sham-exposed mice, but the group difference was not statistically significant.

Water consumption was approximately 4 g per day and mouse, and not different between exposed or sham-exposed animals (data not shown). This value is in accordance to the water intake measurements published by the Jackson Laboratory [31], and obviously not influenced by the experimental set-up.

### Survival and incidence of lymphoma

**Figure 6:**
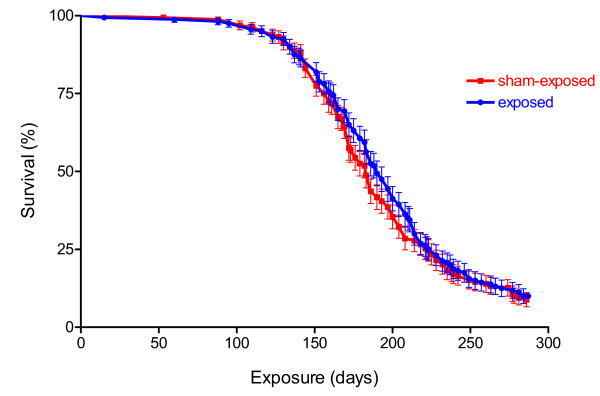
**Survival rates in AKR/J mice exposed to 900 MHz electromagnetic fields. **No significant differences in the survival proportion or mean survival time were seen between exposed and sham-exposed animals when average whole body specific absorption rates were 0.4 W/kg. Data are given as % of 160 animals ± standard error of the mean.

**Figure 7:**
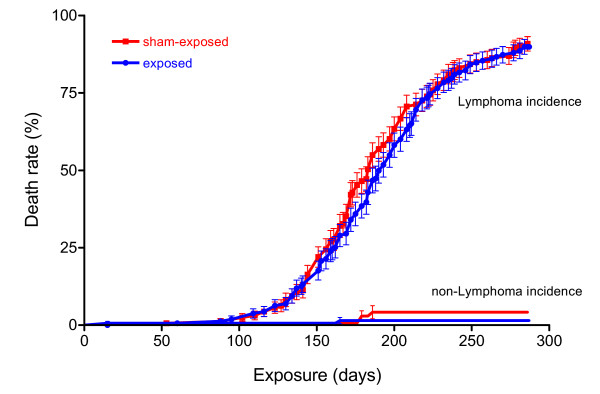
**Lymphoma incidence in AKR/J mice. **Essentially all mortality observed in AKR/J mice was related to the development of hematopoietic diseases independent of electromagnetic field or sham-exposure. Median time for lymphoma development was 183 days (exposed mice) or 193 days (sham-exposed mice), and not significantly different according to the logrank test, with animals censored which were still alive at the end of the study or died of other reasons than lymphoma. Data are given as % of 160 animals ± standard error of the mean.

**Figure 8:**
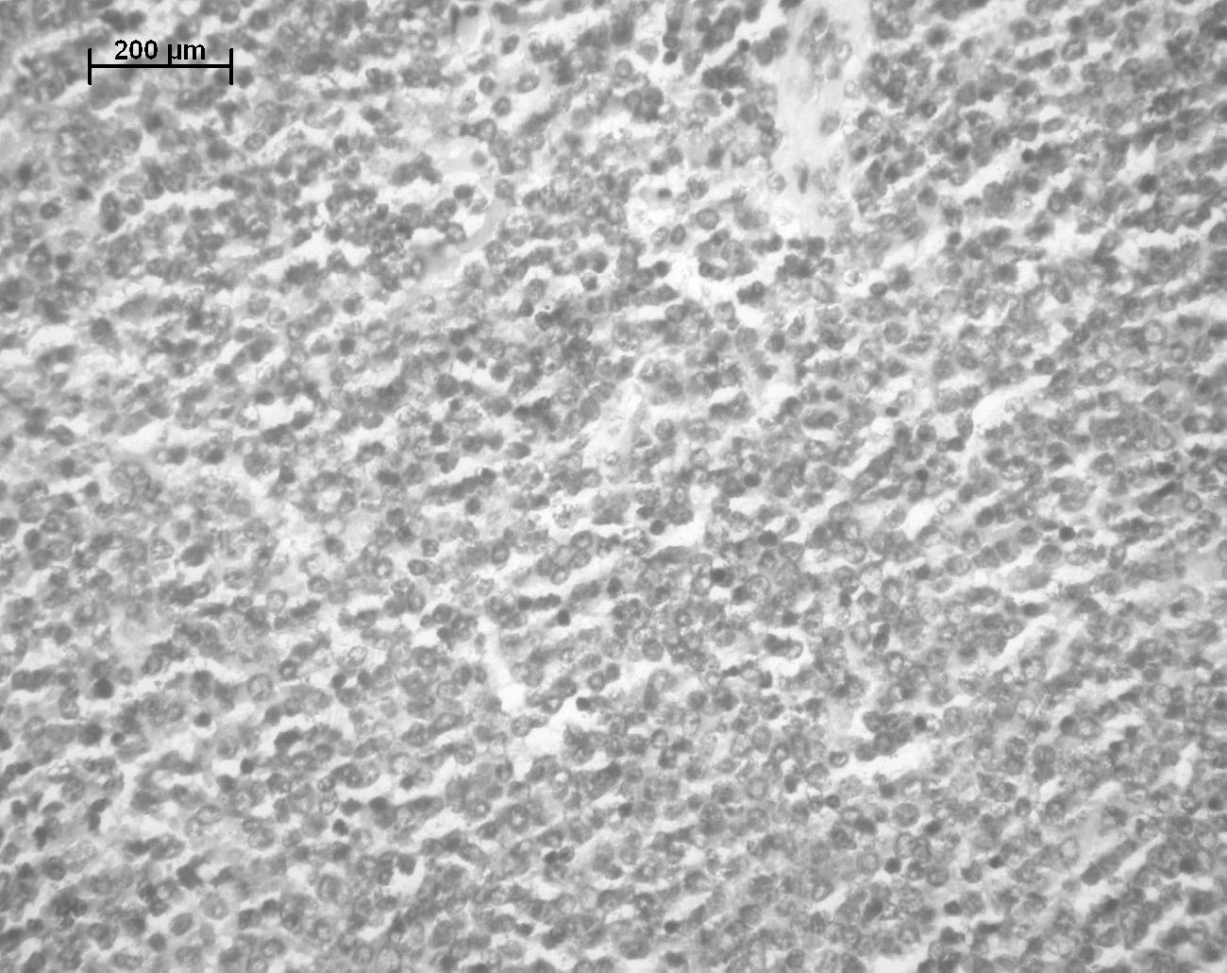
**Lymphoblastic lymphoma in AKR/J mice. **The architecture of spleen (seen here) or other involved organs was not maintained due to the development of lymphoblastic lymphoma. Often starry-sky appearance was present and mitotic figures were numerous.

Similar survival rates were seen in both groups of AKR/J mice (Figure 6). The first exposed mouse died of lymphoma after 60 days and the first sham-exposed animal after 88 days. Median survival time was 183 (sham-exposed) and 190 days (exposed mice), and not significantly different according to the logrank test, with animals censored which were still alive at the end of the study. Patterns of tumor-related mortality in the sham-control group were consistent with those observed in a previous study conducted in this laboratory with AKR/J mice [32]. As seen in our previous study, essentially all mortality observed in AKR/J mice was related to the development of lymphoblastic lymphomas [33,34] (Figures 7, 8). Exceptions were 3 animals with rectal eczema (2 exposed, 1 sham-exposed), one animal with unclear findings and two sham-exposed animals with protruded vagine. These findings were considered random findings and not related to the exposure.

### Clinical picture

**Figure 9:**
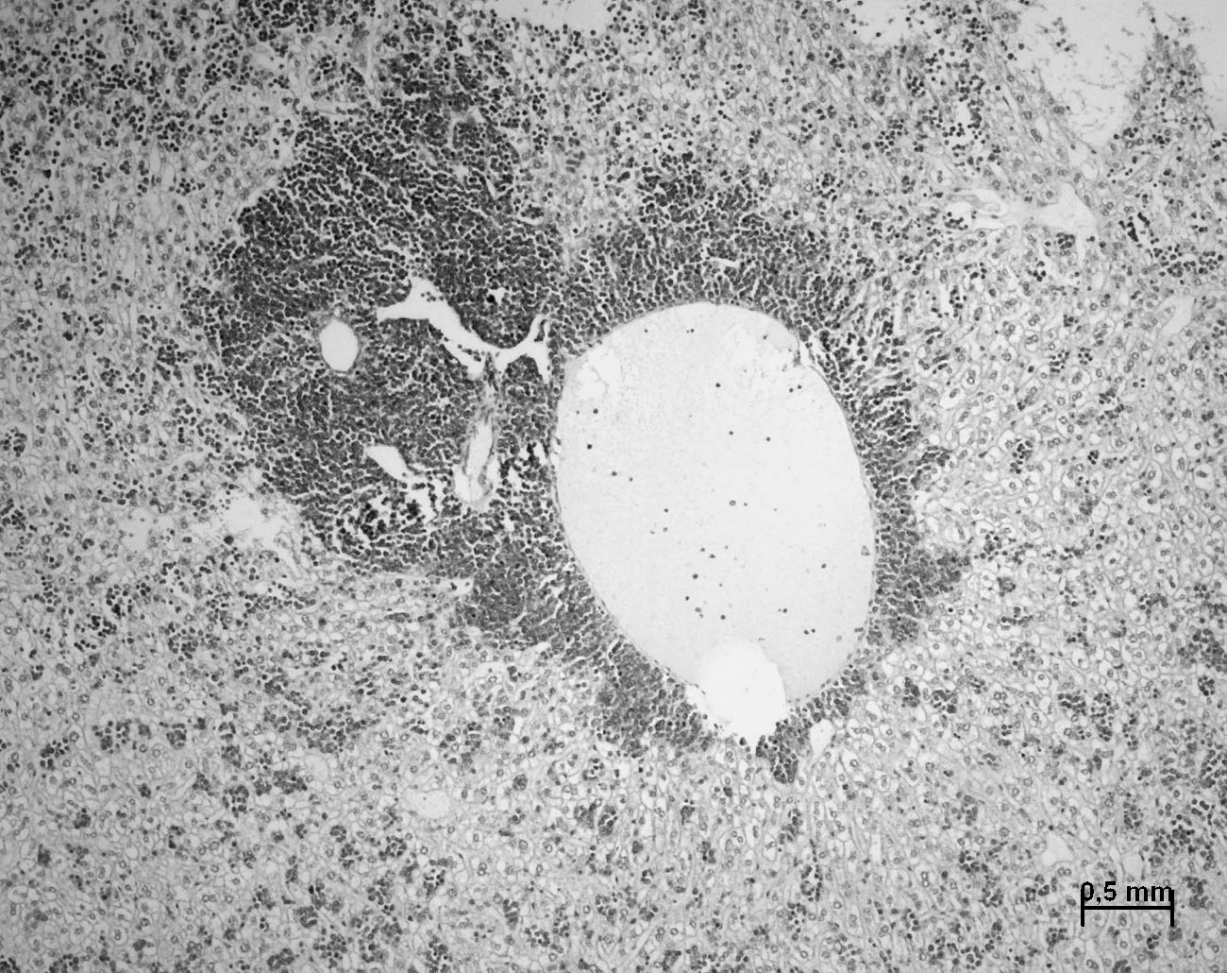
**Malignant lymphoma metastatic to liver in AKR/J mice. **Extensive proliferation of lymphoma cells around portal tracts was seen in liver. Infiltrations resulted in few cases in macroscopically visible tumors in liver or spleen of up to 1 cm in diameter.

In female AKR/J mice lymphoma developed rapidly, usually associated with lymphadenopathy. In 28.2% (exposed) and 30.3% (sham-exposed) of all animals, lymphomas were restricted to the thymus, followed by respiratory distress and protrusion of the eyes. 13 animals (8 exposed, 5 sham-exposed) died in their cages without any earlier sign of distress, although autopsy revealed enlarged mediastinal mass compressing the thoracic space. The lung was affected in 10% of the exposed and sham-exposed animals; 8 exposed and 6 sham-exposed animals developed macroscopically visible metastatic tumors in the liver or spleen (Figure 9). Other clinical observations like splenomegaly and ruffled fur were considered to be associated with neoplastic development and did not correlate with the electromagnetic field exposure. Tumors of other sides such as mammary gland or intestine could not been observed.

**Figure 10:**
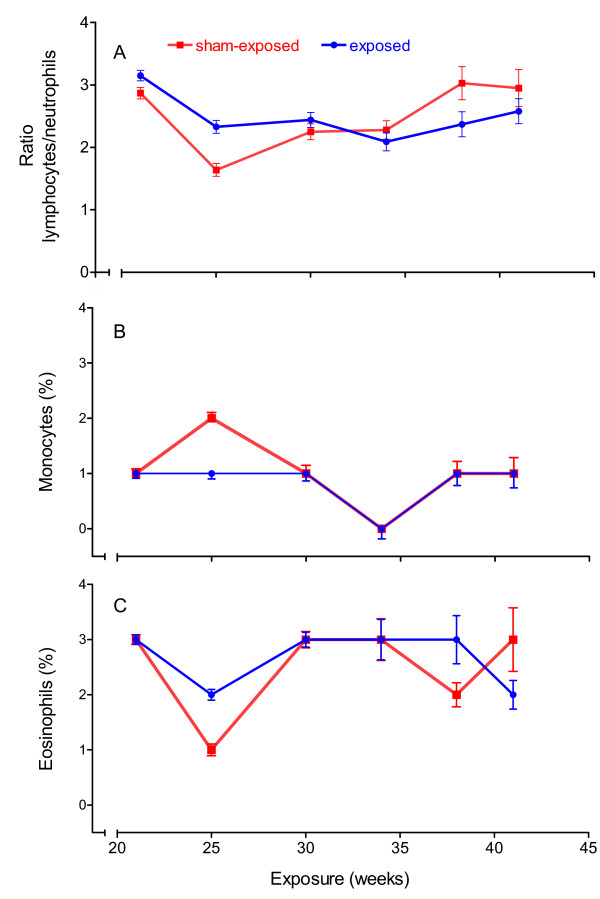
**Differential leucocyte count. **Differential leucocyte counts of peripheral blood of healthy female AKR/J mice did not differ significantly between animals sham-exposed or exposed to 900 MHz electromagnetic fields, although time influenced the ratio of lymphocytes to neutrophilic granulocytes (A) as well as the percentage of monocytes (B) and eosinophilic granulocytes (C). Percentage of basophilic granulocytes was in all cases below 1. 100 cells were counted per slice. Mean ± standard error of the mean, n = 138 (exposed) or 137 (sham-exposed) after 21 weeks of exposure.

When the animals reached an age of about half a year, differential counts of leucocytes were performed monthly using blood smears from tail blood. As seen in Figure 10, exposure to GSM-modulated electromagnetic fields had neither influence on the ratios of lymphocytes to neutrophilic granulocytes nor on counts of monocytes, eosinophilic or basophilic granulocytes. However, the development of lymphoma was associated with changes in the red and white blood picture. Symptoms were changes in the counts of lymphocytes and neutrophilic granulocytes, toxic granulation in neutrophilic granulocytes, the occurrence of juvenile cells or blasts, blastoid or atypical lymphocytes, Gumprecht's shadows, leucopenia, anisocytosis, poikilocytosis or enhanced polychromasia. These changes were not related to the exposure.

## Discussion

The present study was designed to test the hypothesis that 900 MHz electromagnetic fields, modulated according to the global system for mobile communication (GSM), increases the risk of lymphoma incidence in female AKR/J mice. According to the results this hypothesis has to be rejected, since compared with sham-exposed females of this strain, a mean exposition of 0.4 W/kg SAR neither influenced the risk to develop lymphoma, nor the malignancy of the disease, only an influence on the growth pattern of the animals was observed. However, the present experiment does not allow any conclusions about tumor onset or the kinetics of tumor development, since for such type of study animals would have to be sacrificed and examined at fixed intervals irrespective of clinical symptoms.

Exposure to electromagnetic fields increased the growth rate in the nematode *Caenorhabditis elegans *[35], but decreased the birth weight of albino rat offsprings [36]. In contrast, the growth pattern of Eμ-*Pim*1 mice was not changed due to exposure to electromagnetic fields [17,18]. During the progression of the present experiment, female AKR/J mice showed a tendency to obesity. Accumulation of weight was significantly related to the exposition (see Figure 5), leading to a higher body weight gain in exposed compared with sham-exposed mice.

Food was available *ad libitum*, and various studies described effects of electromagnetic fields on the nervous system in humans or rodents [37-39]. It may, therefore, be possible that the exposure to the electromagnetic field influenced the appetite, leading to higher food intake. However, the exposure-independent water consumption does not indicate that major changes in the intake of food occurred.

Mitochondrial heat production is one of the main energy consuming processes in endotherms like mice [40,41]. The Interaction of electromagnetic fields with water molecules in cells results also in heat production. Although the continuous exposure in our study should not have increased the mice's body temperatures, it may have contributed in keeping the animal's body temperature above ambient, therewith economizing mitochondrial heat production. If mitochondrial functions were not changed due to the electromagnetic field exposure, unchanged food intake may so have let to increased fat accumulation. In this context it is important to compare the SAR value of 0.4 W/kg with the total energy consumption of mice (approximately 5 W/kg), thus on the order of 10%. Since the electromagnetic energy is absorbed passively (i.e., not originating from metabolizing food), the increased body weight might be a consequence of a shift in energy utilization. This hypothesis, however, must be examined by specific studies of the mice' metabolism. Anyhow, such an effect would be visible only in long-term exposure studies and probably insignificant with only 1 hour exposure per day [17,18].

A demonstration that long-term exposure to electromagnetic fields derived from mobile phones or base stations increases the incidence of tumors in animals would provide direct evidence that such radiation is carcinogenic. The most positive evidence of an effect of exposure to high frequency electromagnetic fields similar to that used by mobile phones was reported by Repacholi et al. [17], using Eμ-*Pim*1 transgenic mice, which are known to develop spontaneous lymphoma with a high incidence rate. Lymphoma risk was found to be significantly higher in the exposed Eμ-*Pim*1 mice than in the controls, mostly pronounced for non-lymphoblastic lymphoma. Humans are presently not known to carry an activated *Pim*1 gene, but other inherited gene defects are known that predispose carriers to develop cancer [19]. However, an investigation within the electromagnetic energy program of the National Health and Medical Research Council of Australia, using the same Eμ-*Pim*1 mice from the same supplier (Taconic Farms, New York) as Repacholi et al. [17], did not find a significant effect of 898.4 MHz GSM radiofrequency radiation at SAR values of up to 4.0 W/kg [18]. Because of the importance of these studies, a replication started according to plan in spring 2002 in Italy. First results are expected 2005. Nevertheless, the inconsistent results already indicated the need of further assessment of the relevance of such findings for human health [10].

A Japanese study showed that neither 1.5 GHz nor 929.3 MHz electromagnetic field exposure promotes liver carcinogenesis in a medium-term bioassay system, using partially hepatectomised F344 rats [42]. A monopole antenna close to the constrained animals emitted a "near-field" radiation that resulted in SAR values of 0.45–0.68 W/kg as a whole-body average and of 0.9–1.9 W/kg in the liver. It was suggested that the applied "near-field", which is more in line with the actual exposure conditions of cellular telephone users, explains the differences to the study of Repacholi et al. [17], who employed a "far-field" and mean whole-body SAR values of 0.13–1.4 W/kg. However, the confinement may also affect the results [43,44], as well as the different animal models: long-term exposure of genetically predisposed animals on the one hand and a medium-term bioassay of chemically induced carcinogenesis on the other hand. Therefore, it is difficult to decide which results are more relevant for human mobile phone users.

In the present study non-restrained mice that are genetically predisposed to develop lymphoma with a high incidence were exposed to a "far-field" similar to both Australian studies [17,18], with the difference that our mice were exposed for 24 h, whereas their mice were exposed for 1 hour per day only. In contrast to Repacholi et al. [17], we could not observe an increased lymphoma risk. However, our study's results are consistent with the investigation within the electromagnetic energy program of the National Health and Medical Research Council of Australia [18]. The authors also did not find a significant effect of 898.4 MHz GSM radiofrequency radiation at SAR values of up to 4.0 W/kg when compared to sham-irradiated animals. The overall conclusion of the present study as well as of data from the literature is that they lack evidence that electromagnetic field exposure increases the incidence of leukaemia in rodents.

## Conclusions

The present study does not support the hypothesis that chronic exposure to high-frequency electromagnetic fields, similar to those emitted by mobile phones and base stations promote neoplastic development in the hematopoietic system in genetically predisposed mice. However, we cannot rule out that exposure to electromagnetic fields may be risk factors for other neoplastic development.

## Competing interests

The authors declare that they have no competing interests.

## Authors' contributions

AS carried out the study, performed the statistical analyses and drafted most of the manuscript. AL conceived the study, participated in its design and coordination, and drafted some parts of the manuscript. JS, AB and VH developed the technical set up and delivered the dosimetry for the experiment. All authors read and approved the final manuscript.

## Pre-publication history

The pre-publication history for this paper can be accessed here:
